# Atorvastatin and ezetimibe protect against hypercholesterolemia-induced lung oxidative stress, inflammation, and fibrosis in rats

**DOI:** 10.3389/fmed.2022.1039707

**Published:** 2022-11-24

**Authors:** Porrnthanate Seenak, Sarawut Kumphune, Thanit Prasitsak, Nitirut Nernpermpisooth, Wachirawadee Malakul

**Affiliations:** ^1^Integrative Biomedical Research Unit (IBRU), Faculty of Allied Health Sciences, Naresuan University, Phitsanulok, Thailand; ^2^Department of Cardio-Thoracic Technology, Faculty of Allied Health Sciences, Naresuan University, Phitsanulok, Thailand; ^3^Biomedical Engineering Institute (BMEI), Chiang Mai University, Chiang Mai, Thailand; ^4^Department of Oral Biology, Faculty of Dentistry, Naresuan University, Phitsanulok, Thailand; ^5^Department of Physiology, Faculty of Medical Sciences, Naresuan University, Phitsanulok, Thailand

**Keywords:** atorvastatin, ezetimibe, hypercholesterolemia, lung oxidative stress, lung inflammation, lung fibrosis

## Abstract

**Background:**

Hypercholesterolemia is a major risk factor for interstitial lung disease (ILD). Atorvastatin and ezetimibe are antilipemic drugs that have pleiotropic effects. However, their effects on pulmonary fibrosis prevention and the mechanisms underlying hypercholesterolemia have not been fully investigated. This study aimed to evaluate the individual effects of atorvastatin and ezetimibe on lung inflammation and fibrosis in high-cholesterol diet (HCD)-fed rats.

**Materials and methods:**

Male Sprague-Dawley rats were divided into four groups — standard diet (S), standard diet + 1% cholesterol (SC), standard diet + 1% cholesterol with 30 mg/kg/day atorvastatin (SCA), and standard diet + 1% cholesterol with 10 mg/kg/day ezetimibe (SCE). At the end of an 8-week dietary schedule, serum lipid parameters and the levels of lung oxidative stress, inflammatory cytokines, and fibrotic mediators were determined.

**Results:**

Atorvastatin and ezetimibe treatment remarkably reduced serum lipid profiles with reversed pulmonary histological alterations, in addition to reducing the levels of lung oxidative stress, inflammation, and fibrosis in hypercholesterolemic rats.

**Conclusion:**

Atorvastatin and ezetimibe treatment showed a protective effect against hypercholesterolemia-induced pulmonary fibrosis in rats. This information appears potentially useful in the prevention of PF in a hypercholesterolemia model; however, further rigorous investigations are needed to prove their clinical utility on antifibrosis.

## Introduction

Pulmonary fibrosis (PF) is a chronic interstitial lung disease (ILD) characterized by scarring and degradation of the lung architecture of alveolar–capillary membranes (1, 2). The risk factors for developing PF include smoking, pollution, infection, and dyslipidemia (3–5). However, the etiopathogenesis of PF remains unclear. Hypercholesterolemia is the most common lipoprotein-related disorder, typically presenting with an increase in both plasma triglyceride and low-density lipoprotein cholesterol (LDL-C) levels and a decrease in high-density cholesterol (HDL-C) (6, 7). Patients with PF often have reduced plasma HDL-C levels compared to healthy individuals (8, 9). Lipids and lipoproteins are hypothesized to play roles in the early stages of pathogenesis of interstitial lung injury, inflammation, and fibrosis (4). HDL-C exerts beneficial effects in subclinical ILD by attenuating lung inflammation, inducing extracellular matrix remodeling, and altering surfactant function (4). Cholesterol-lowering drugs not only contribute to the regulation of plasma lipoproteins but also exert extra-lipid effects. Recently, statins have received increased attention for their potential use in the treatment of idiopathic pulmonary fibrosis (IPF) (3). However, some studies have reported contradictory findings that statin use aggravates PF in smokers (10).

The combined simvastatin and ezetimibe therapy reportedly results in a significant reduction in secretion of the pro-inflammatory cytokine IL-1β from monocytes in hypercholesterolemia patients (11). Additionally, ezetimibe was shown to preserve collagen content during plaque inflammation in atherosclerosis and influence the activity of the central pro-inflammatory regulator nuclear factor-κB (NF-κB) (12). Thus, each hypolipemic drug has unique pleiotropic properties. However, it is unclear whether different types of lipid-lowering drugs, including atorvastatin and ezetimibe, can modulate lung inflammation and extracellular matrix remodeling, which can be potentially useful in the prevention of PF in a hypercholesterolemia model. Therefore, the current study aimed to determine the effects of atorvastatin and ezetimibe therapy on lung oxidative stress, pro- and anti-inflammatory factors, and matrix metalloproteinase (MMP) homeostasis and the molecular mechanisms underlying PF in hypercholesterolemic rats.

## Materials and methods

### Animals and diets

Experiments with Sprague-Dawley rats were approved by the Animal Ethics Committee of Naresuan University, Thailand (approval number: NU-AE620718). The use of rats was in accordance with institutional guidelines for the care and use of laboratory regulations. Four-week-old male rats (150–200 g) were obtained from Nomura Siam International Co., Ltd. (Bangkok, Thailand) and housed in temperature-controlled conditions (25 ± 2°C) under a 12-h light–dark cycle with free access to water and normal chow for one week at the Centre for Animal Research, Naresuan University, Phitsanulok, Thailand. The acclimatized rats were randomly allocated to four groups (*n* = 5–6) and fed the following diets for eight weeks: standard diet (S), high-cholesterol diet [standard diet + 1% (w/w) cholesterol; SC], SC with atorvastatin (30 mg/kg/day; SCA), and SC with ezetimibe (10 mg/kg/day; SCE). Simvastatin and ezetimibe were administered via oral gavage. Body weight (BW), abdominal circumference (AC), and total food consumption were recorded. Rats were euthanized using pentobarbital sodium (100 mg/kg) and lithium heparin (100 U), followed by lung tissue and blood serum collection.

### Measurement of blood parameters

Total cholesterol (TC), high-density lipoprotein cholesterol (HDL-C), low-density lipoprotein cholesterol (LDL-C) and triglyceride (TG) levels were analyzed using an automated system biochemistry analyzer (Cobas c 111 analyzer, Basel, Switzerland).

### Determination of cytokine levels in the lungs

Lung cytokine levels were analyzed using an enzyme-linked immunosorbent assay (ELISA). The levels of interleukin 1 beta (IL1-β; catalog no: 900-M91; Prepotech^®^, USA), interleukin 6 (IL-6; catalog no: 900-M86; Prepotech^®^, USA), interleukin 10 (IL-10; catalog no: ab100765; Abcam, UK), tumor necrosis factor-alpha (TNF-α; catalog no: ab100785; Abcam, UK) and transforming growth factor-beta (TGF-β; catalog no: ab119558; Abcam, UK) were determined according to the manufacturers’ protocols.

### Reverse transcription-quantitative polymerase chain reaction

Total RNA from the frozen lung tissues was extracted using TRIzol (Invitrogen, Carlsbad, CA, USA) and the PureLink™ RNA Mini Kit (catalog no: 12183020; Invitrogen, Carlsbad, CA, USA). The Tetro cDNA Synthesis Kit (catalog no: BIO-65043; BIOLINE) was used to synthesize the cDNA. Quantitative RT-PCR was performed using a SensiFAST STBR^®^ No-ROX Kit (catalog no: BIO-98005; BIOLINE) and C1000 Touch™ Thermal Cycler (Bio-Rad^®^) with specific primers (Table 1). Relative gene expression levels were determined using the 2^–^^ΔΔ^ ^Ct^ method.

**TABLE 1:** Primer sequences for quantitative reverse transcription-PCR.

Target genes	Forward primer	Reverse primer	Reference
β-Actin	5′-CCCGCGAGTACAACCTTCT- 3′	5′CGTCATCCATGGCGAACT- 3′	(13)
**Pro-fibrotic enzymes**			
*MMP-3*	5′-GAGAACTTTCCAGGCATTGG- 3′	5′CCGCTGAAGAAGTAAAGAAACC- 3′	(13)
*MMP-7*	5′ -CGGCGGAGATGCTCACTTT- 3′	5′ -GCCAAGTTCATGAGTGGCAAC- 3′	(14)
*MMP-9*	5′ -CTTTGTAGGGTCGGTTCTG- 3′	5′ -TGGTGTCCTCCGATGTAAG- 3′	(15)
*MMP-13*	5′-GGGACGCCCATTTTGATG-3′	5′-AGCTCATGGGCAGCAACAAT-3′	(16)
**Anti-fibrotic enzymes**			
*MMP-1*	5′ -ACAGTTCCCCGTGTTTCAG- 3′	5′ -CCCACACCTAGGTTTCCTCA- 3′	(15)
*MMP-19*	5′-GGAAACAAGGTGTGGCGGTAT-3′	5′-CATCTAGGTTGGGTTCCACTCTGT-3′	(17)

### Histology, oil red O Staining, and oxidative stress detection

For determining histological structure and fibrosis, the lungs were fixed with 10% (v/v) formalin for 24 h. The fixed lungs were embedded in paraffin and sectioned at 5 μm. The lung sections were then rehydrated in a graded ethanol series, followed by hematoxylin and eosin (H&E) and Masson’s trichrome staining. The percentage of collagen area was analyzed by imageJ software following the formula: collagen area (%) = (collagen area/total image area) × 100. To evaluate fat accumulation in the lungs, frozen lung tissues were embedded and sectioned at the optimum cutting temperature (catalog no: 05-9801; Bio-optical, Milano, Italy), followed by staining and counterstaining with an oil red O working solution (catalog no: O0625; Sigma-Aldrich, USA) and hematoxylin, respectively.

For oxidative stress detection, frozen lung sections were stained with dihydroethidium (catalog no: D7008; Sigma-Aldrich, USA). All specimens were captured under a light or fluorescence microscope (ZEISS^®^ Axio observer D1 connected to AxioCam MRc5) and analyzed using Zen pro software.

### Immunofluorescence staining

Lung sections were sectioned at 5 μm thickness, deparaffinized, and rehydrated in a graded ethanol series. Antigen retrieval was performed using a citrate buffer solution, followed by incubation with 2% normal goat serum (Jackson ImmunoResearch Laboratory, Inc., West Grove, PA, USA) and rabbit anti-TGFβRII primary antibody (catalog no: ab18683; Abcam, 1:200, UK). Sections were then incubated in the Alexa Fluor 488-conjugated goat anti-rabbit IgG secondary antibody (catalog no: ab150085; Abcam, UK) and DAPI (catalog no: D9542; Sigma-Aldrich, USA) for immunofluorescence detection. Images were captured using a slidescanner (ZEISS^®^ Axio Scan. Z1), connected to an LED light source (ZEISS^®^ Colibri 7), with an imaging software (ZEN Blue 2.3).

### Western blot analysis

Lung proteins were extracted, separated using 12% SDS-PAGE, and subsequently transferred onto polyvinylidene difluoride (PVDF) membranes. The membranes were blocked for 1 h in a blocking solution [5% non-fat milk in Tris-buffered saline (pH 7.4)] and incubated with the following primary antibodies overnight at 4^°^C: anti HO-1 (catalog no: ab13243; Abcam, 1:1,000, UK), anti p-smad2/3 (catalog no: 8828; Cell Signaling Technology, 1:1,000, USA), and anti t-smad2/3 (catalog no: 8685; Cell Signaling Technology, 1:1,000, USA). The membranes were washed and incubated with a horseradish peroxidase (HRP)-conjugated secondary antibody (catalog no: ab8227; Abcam, 1:5,000, UK) for 2 h at 25^°^C (room temperature). Antibody signals were determined using an HRP detection reagent (catalog no: WBLUF0100; Merck Millipore, Germany) and detected using the Gel Doc XR + system (Bio-Rad Laboratories, Inc., Hercules, CA, USA). Image Lab software version 5.2.1 (BioRad Laboratories, Inc., Hercules, CA, USA) was used to evaluate band densities, which were normalized to the expression of actin as a housekeeping protein.

### Statistical analysis

Results are presented as mean ± SEM. A one-way analysis of variance (ANOVA) with Tukey’s *post hoc* analysis was performed using GraphPad Prism 5.0. Statistical significance was set at *p* < 0.05.

## Results

### Model characteristics and lipid parameters

**FIGURE 1:**
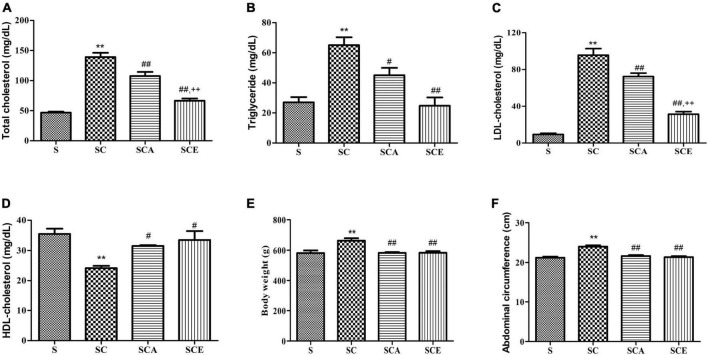
Effects of atorvastatin and ezetimibe treatment on lipid parameters and model characteristics in hypercholesterolemic rats. **(A)** Total cholesterol (TC); **(B)** triglyceride (TG); **(C)** LDL-cholesterol (LDL-C); **(D)** HDL-cholesterol (HDL-C); **(E)** body weight (BW) and **(F)** abdominal circumference (AC). Data are presented as mean ± SEM. **p < 0.01 vs. the control group; #*p* < 0.05; ##*p* < 0.01 vs. the SC group; ++*p* < 0.01 vs. the SCA group (*n* = 6 per group).

The characteristics of the rat model are presented in Figure 1. The SC group had significantly higher final body weight and abdominal circumference than the control group (*p* < 0.05). Treatment with atorvastatin and ezetimibe for eight weeks significantly reduced the final body weight (583.2 ± 4.95 g and 583.6 ± 10.86 g vs. 663.5 ± 16.37 g, respectively, *p* < 0.01) and abdominal circumference when compared to those in the SC group (21.64 ± 0.23 cm and 21.36 ± 0.26 cm vs. 24 ± 0.32 cm, respectively, *p* < 0.01) (Figures 1E,F), while the BMI remained unaltered in all groups.

Lipid profile assessment revealed that the high cholesterol diet-fed rats had significantly increased TC, TG, and LDL-C levels compared to the control group (*p* < 0.01) (Figures 1A–C). The SCA and SCE groups showed significant reduction in TC (107.5 ± 6.83 mg/dL and 66.50 ± 3.8 mg/dL vs. 139 ± 7.31 mg/dL, *p* < 0.01), TG (45 ± 4.97 mg/dL and 24.75 ± 5.57 mg/dL vs. 65 ± 5.27 mg/dL, *p* < 0.01) and LDL-C (72.33 ± 3.72 mg/dL and 31.40 ± 2.76 mg/dL vs. 95.67 ± 7.11 mg/dL, *p* < 0.01) levels compared to the SC group (Figures 1A–C). Both SCA and SCE groups showed significantly higher HDL-C levels than the SC group (31.50 ± 0.28 mg/dL and 33.50 ± 2.9 mg/dL vs. 24.17 ± 0.74 mg/dL, *p* < 0.01) (Figure 1D).

### Atorvastatin and ezetimibe treatment reverses hypercholesterolemia-induced lung histopathological alterations and fat accumulation

**FIGURE 2:**
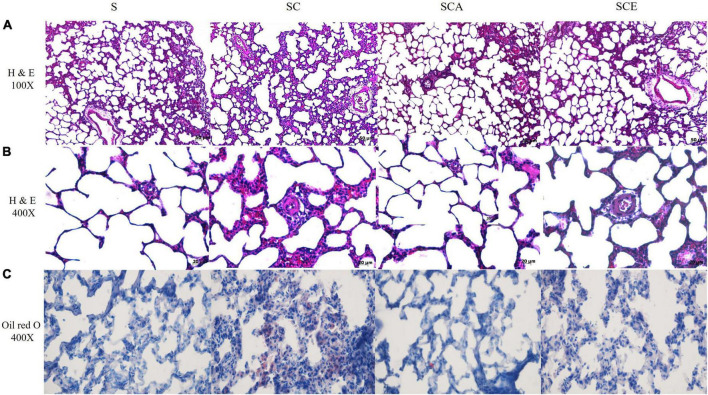
Effects of atorvastatin and ezetimibe treatment on lung histopathological alterations and fat accumulation in hypercholesterolemic rats. **(A)** Lung H&E staining, 100×; **(B)** lung H&E staining, 400×; **(C)** oil red O staining, 400×.

We verified the effects of hypercholesterolemia on lung morphological changes and lipid droplet accumulation by staining rat lungs with H&E and oil Red O. An SC diet resulted in inflammatory cell infiltration, increased alveolar wall thickness, and reduced alveolar spacing compared to those in the control group. Oil Red O staining revealed lipid droplet accumulation in the alveolar septa in the SC group. Treatment with atorvastatin and ezetimibe prevented histopathological alterations and lipid droplet accumulation in hypercholesterolemic rat lungs (Figures 2A–C).

### Atorvastatin and ezetimibe treatment reverses the lung oxidative stress caused by hypercholesterolemia

**FIGURE 3:**
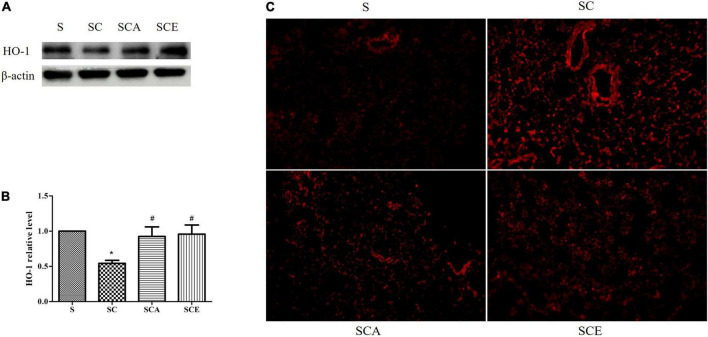
Effects of atorvastatin and ezetimibe treatment on lung oxidative stress in hypercholesterolemic rats. **(A)** Western blot bands of heme oxygenase-1 (HO-1) and Beta-actin (β-actin) expression in lung tissues; **(B)** quantified levels of lung HO-1 expression; **(C)** lung dihydroehthidium (DHE) staining.

Oxidative stress was determined by staining rat lungs with DHE and determining the expression of heme oxygenase (HO)-1. As shown in Figure 3, the groups with hypercholesterolemia showed significantly increased DHE staining and downregulated HO-1 protein expression. Hypercholesterolemia-induced oxidative stress in lung tissue was attenuated by atorvastatin and ezetimibe treatment (Figures 3A–C). These results indicate that atorvastatin and ezetimibe treatments prevent hypercholesterolemia-induced lung oxidative stress by upregulating antioxidant defense mechanisms.

### Atorvastatin and ezetimibe treatment improves the homeostasis of pro-inflammatory and anti-inflammatory cytokines in rat lungs with hypercholesterolemia

**FIGURE 4:**
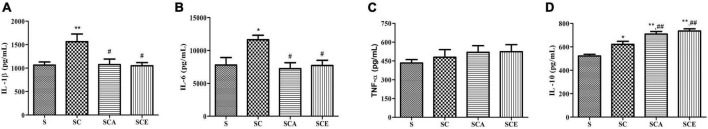
Effects of atorvastatin and ezetimibe treatment on the levels of lung pro-inflammatory and anti-inflammatory cytokines in high-cholesterol fed rats. **(A)** Tumor necrosis factor-alpha (TNF-α); **(B)** interleukin-6 (IL-6); **(C)** interleukin-1 beta (IL-1β); **(D)** interleukin-10. Each bar represents mean ± SEM. *p < 0.05; **p < 0.01 vs. the control group; #*p* < 0.05; ##*p* < 0.01 vs. the SC group (*n* = 6 per group).

The balance between pro-inflammatory and anti-inflammatory cytokine levels is essential for lung homeostasis. We determined the levels of pro-inflammatory cytokines, including IL-1β, IL-6, and TNF-α, as well as IL-10, an anti-inflammatory cytokine, in rat lungs. We observed a significant increase in the levels of both pro- and anti-inflammatory cytokines in rat lungs from the SC group compared to that in the control group — IL-1β (1563 ± 169.4 ng/mL vs. 1064 ± 66.73 ng/mL, *p* < 0.01), IL-6 (11628 ± 678.3 ng/mL vs. 7781 ± 1139 ng/mL, *p* < 0.05), and IL-10 (621.9 ± 25.96 ng/mL vs. 522.6 ± 13.57 ng/mL, *p* < 0.05) (Figures 4A,B,D). Treatment with atorvastatin and ezetimibe significantly reduced the levels of the pro-inflammatory cytokines IL-1β (1076 ± 116.5 and 1051 ± 67.73 ng/mL vs. 1563 ± 169.4 ng/mL, *p* < 0.05) and IL-6 (7226 ± 905.8 ng/mL and 7721 ± 770.1 ng/mL vs. 11628 ± 678.3 ng/mL, *p* < 0.05) and significantly increased the levels of the anti-inflammatory cytokine IL-10 compared to those in the SC group (709.8 ± 21.91 and 735.9 ± 18.15 ng/mL vs. 621.9 ± 25.96 ng/mL, *p* < 0.05) (Figures 4A,B,D). There were no significant differences in TNF-α levels (Figure 4C). These results indicate that atorvastatin and ezetimibe treatment can restore the balance between pro-inflammatory and anti-inflammatory cytokines in the lungs of hypercholesterolemic rats.

### Atorvastatin and ezetimibe treatment reverses the hypercholesterolemia-activated transforming growth factor-beta/mothers against decapentaplegic homolog signaling pathway

**FIGURE 5:**
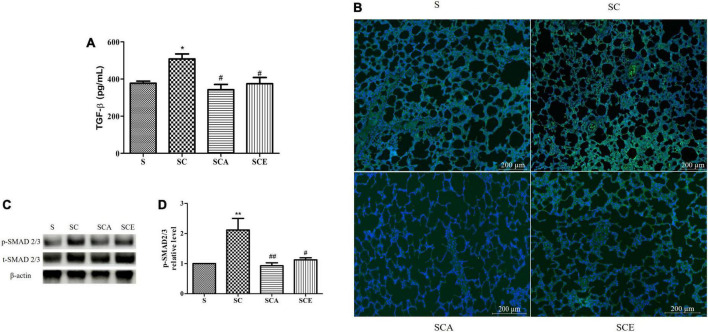
Effects of atorvastatin and ezetimibe treatment on lung TGF-β/Smad signaling pathway in high-cholesterol fed rats. **(A)** Transforming growth factor-beta (TGF-β); **(B)** transforming growth factor-beta receptor (TGF-β R) immunofluorescence staining; **(C)** western blot bands of phosphorylated Smad2/3 (p-Smad2/3), total Smad2/3 (t-Smad2/3) and beta-actin (β-actin) expression in lung tissues; **(D)** quantification of lung p-Smad2/3 expression. Data are presented as mean ± SEM. *p < 0.05; **p < 0.01 vs. the control group; #*p* < 0.05; ##*p* < 0.01 vs. the SC group (*n* = 6 per group).

The TGF-β/SMAD-dependent signaling pathway plays a crucial role in fibrotic lung scarring. Here, we determined the protein expression of TGF-β and p-SMAD2/3 and activation of the TGF-β receptor. Induction of hypercholesterolemia caused an increase in the expression of TGF-β (508.1 ± 27.23 ng/mL vs. 377.3 ± 11.49 ng/mL, *p* < 0.05) and TGF-β receptor compared to that in the control group (Figures 5A,B). Moreover, the SC group had significantly higher p-SMAD2/3 levels than the control group (Figures 5C,D). Treatment with atorvastatin and ezetimibe significantly prevented activation of the TGF-β/SMAD signaling pathway compared with that in the SC group. These results indicate that atorvastatin and ezetimibe have an inhibitory effect on the progression of lung fibrosis.

### Atorvastatin and ezetimibe treatment reverses the hypercholesterolemia-induced development of lung fibrosis

**FIGURE 6:**
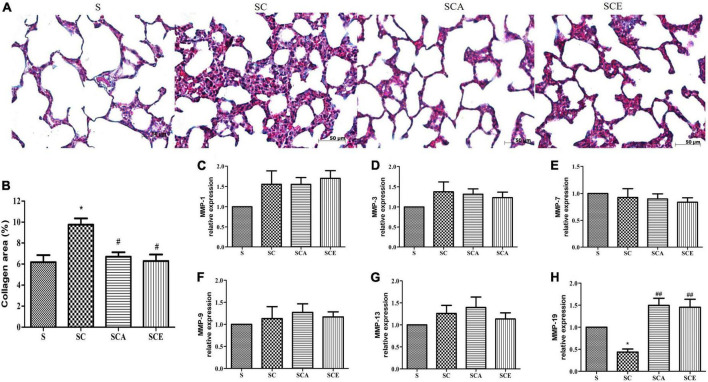
Effects of atorvastatin and ezetimibe treatment on lung fibrosis and matrix metalloprotease enzyme (MMP) expression in hypercholesterolemic rats. **(A)** Masson’s trichrome staining; **(B)** percentage of Masson’s trichrome stained-area; **(C)** MMP-1 expression; **(D)** MMP-3 expression; **(E)** MMP-7 expression; **(F)** MMP-9 expression; **(G)** MMP-13 expression; **(H)** MMP-19 expression. Each bar represents mean ± SEM. *p < 0.05 vs. the control group; #*p* < 0.05; ##*p* < 0.01 vs. the SC group (*n* = 6 per group).

Hypercholesterolemia leads to oxidative stress and lung inflammation, resulting in the development of fibrosis. We performed Masson’s trichrome staining to evaluate lung fibrosis and observed a significant increase in the levels of positive markers of lung fibrosis and the percentage of Masson’s trichrome-stained area in hypercholesterolemic rats compared with those in the control lungs (9.75 ± 0.6% vs. 4.63 ± 0.66%, *p* < 0.05). In contrast, this increase was not observed in the SCA and SCE groups. Treatment with atorvastatin and ezetimibe significantly reduced the percentage of Masson’s trichrome-stained area when compared to that in the SC group (6.69 ± 0.42% and 6.29 ± 0.61% vs. 9.75 ± 0.6%, *p* < 0.05) (Figures 6A,B). We performed quantitative RT-PCR analysis to determine the levels of pro-fibrotic and anti-fibrotic genes, including *MMP-1*, *MMP-3*, *MMP-7*, *MMP-9*, *MMP-13*, and *MMP-19*. The SC group had significantly lower levels of MMP-19 mRNA than the other groups, whereas there was a significant increase in *MMP-19* mRNA expression in both the SCA and SCE groups (*p* < 0.01) (Figure 6H). There was no significant change in the mRNA levels of *MMP-1*, *MMP-3*, *MMP-7*, *MMP-9*, and *MMP-13* (Figures 6C–G). These findings suggest that hypercholesterolemia results in the suppression of anti-fibrotic gene expression and that treatment with atorvastatin and ezetimibe inhibits lung fibrosis by enhancing anti-fibrotic gene expression.

## Discussion

A high-cholesterol diet (HCD) is often associated with lung injury. Our findings highlight the lipid-lowering and pleiotropic effects of atorvastatin and ezetimibe treatment on the balance of inflammation and anti-oxidative stress. Both the drugs completely attenuated hypercholesterolemia-induced lipid accumulation and the development of lung fibrosis.

Hyperlipidemia is a known major cause of increased LDL-C and TG levels and induces visceral fat accumulation, leading to obesity (18). We showed that rats fed an HCD had significantly increased abdominal circumference, LDL-C, TG, and BW. Treatment with atorvastatin and ezetimibe can attenuate high-fat diet-induced obesity by lowering lipid serum levels via reduced hepatic lipogenesis (19) and reduce the intestinal absorption of cholesterol (20), respectively.

Oxidative stress is thought to play a major role in the pathogenesis of PF (21, 22). Hyperlipidemia results in reduced protection against antioxidants and increased reactive oxygen species (ROS) levels (23); however, the potential role of hypercholesterolemia in the pathogenesis of PF has not been widely investigated. Many studies have demonstrated that elevated plasma lipid levels trigger the generation of ROS, which promotes the expansion of adipose tissue via hypertrophy and/or hyperplasia (21–23). These alterations activate the generation of adipokines, which leads to chronic low-grade systemic inflammation (24). This phenomenon can cause injury to end organs, including the heart, kidneys, blood vessels, and especially the lungs (7, 25). Compared to other organs, the lungs are exposed to the highest levels of oxygen, which makes them highly vulnerable to oxidative stress (26). In general, the lungs have several antioxidant defense mechanisms to protect against the harmful effects of ROS (26). A reduction in these defenses contributes to oxidative stress, which may directly damage the lung or indirectly cause injury by reducing antiprotease activity and promoting the degradation of extracellular matrix (ECM) components (14, 22), leading to the progression of lung diseases, including PF (4, 27, 28). Moreover, high levels of circulating LDL-C directly alter lung cholesterol homeostasis, leading to alveolar type II cell injury and cholesterol overloading, which can further induce lung oxidative stress and inflammation (4, 28). Heme oxygenase-1 (HO-1), a potent antioxidant enzyme, plays an important role in defense against oxidative stress, and its potential antioxidant properties also regulate the inflammatory response. Animal studies have demonstrated that HO-1 plays a critical protective role in several lung diseases, including pulmonary hypertension and PF (29). We showed here that atorvastatin and ezetimibe therapy attenuated the hypercholesterolemia-induced lung oxidative stress, as evidenced by the reduced DHE staining and enhanced HO-1 expression. DHE and HO-1 are potent markers of ROS generation and antioxidants, respectively (29, 30). Our results suggest a potential therapeutic role of atorvastatin and ezetimibe in attenuating lung oxidative stress.

Hypercholesterolemia not only induces oxidative stress but also contributes to inflammation, as the oxidative stress resulting from dyslipidemia triggers an imbalance between pro-inflammatory and anti-inflammatory mediators by increasing the expression of numerous cytokines, including IL-1β, IL-6, TNF-α, and IL-10 (31). These changes contribute to inflammatory cell infiltration at the sites of lung injury, with consequent alveolar septa thickening and loss of air spaces (9). Several molecules secreted by inflammatory cells, including hydrogen peroxide, proteases, and phospholipase enzymes, damage lung epithelial cells and cell membrane integrity (32, 33). As observed in the HCD group, hypercholesterolemia causes lung inflammation through elevated IL-1β and IL-6 levels, which are physiologically compensated by increased IL-10 levels. IL-1β and IL-6 are well-known early pro-inflammatory cytokines that induce inflammatory cell infiltration, chronic lung inflammation, and fibrosis, whereas IL-10 plays important anti-inflammatory roles (2). Treatment with atorvastatin and ezetimibe completely prevented HCD-induced lung inflammation and resulted in remarkably higher IL-10 levels than those in the HCD group. Other studies have also reported the efficacy of cholesterol-targeting agents in reducing inflammatory responses in lung disease models (34–37). These observations suggest that atorvastatin and ezetimibe therapy modulates the imbalance between pro-inflammatory and anti-inflammatory cytokines in HCD-fed rats.

IL-1β, IL-6, and lung injury are key active players that lead to a self-perpetuating fibrotic response by directly promoting several cellular mechanisms (27). One potential mechanism of inflammation-induced PF is activation of the canonical TGF-β/SMAD signaling pathway, which promotes dysfunctional ECM production (28). Binding of TGF-β and TGF-β receptor initiates phosphorylation of SMAD2/3, which in turn regulates the gene expression in processes such as cell growth, contraction, and myofibroblast differentiation (31). Moreover, increased TGF-β levels also modulate the balance between pro-fibrotic and anti-fibrotic enzymes and the consequent PF (38). However, the non-canonical pathways related to the effects of atorvastatin and ezetimibe on hypercholesterolemia-induced pulmonary fibrosis, including phosphatidylinositol 3-kinase (PI3K)/Akt, mitogen-activated protein kinases (MAPKs), cell division control protein 42 homolog (CDC42)/Rac, and Rho-like GTPase, as well as follistatin-like 1 (FLST1) need further investigation. Several studies have reported that pro-fibrotic enzymes, including MMP3, MMP7, MMP-9, and MMP-13, can promote epithelial-mesenchymal transition to generate myofibroblast-like cells. Activated myofibroblasts initiate the overproduction of fibronectin, laminin, proteoglycans, and collagen (39, 40). However, these pathological changes are countered by anti-fibrotic enzymes, such as MMP1 and MMP-19, in the resolution stage of the normal response (41). Our results showed that atorvastatin and ezetimibe could prevent HCD-induced lung fibrosis by downregulating the TGF-β-SMAD2/3 signaling pathway and enhancing MMP-19 expression, but not by modulating pro-fibrotic enzyme action. MMP-19 has been reported as a key regulator of PF development (41, 42). It upregulates prostaglandin E2 by enhancing the activity of prostaglandin G/H synthase, which reduces fibroblast migration, proliferation, collagen synthesis, and myofibroblast differentiation (43). This suggests that a disturbance in the anti-fibrotic balance in the lung tissues of HCD-fed rats leads to PF. Interestingly, this is the first study to demonstrate that atorvastatin and ezetimibe treatment can enhance defense mechanisms by upregulating MMP-19. However, the cellular mechanism underlying the atorvastatin and ezetimibe-induced MMP-19 expression needs further investigation. This study demonstrates the pulmoprotective effects of atorvastatin and ezetimibe treatment on hypercholesterolemia-induced PF caused by reducing serum lipid levels and oxidative stress, reversing the balance between pro-inflammatory and anti-inflammatory mediators, downregulating TGF-β-SMAD2/3, and upregulating MMP-19. These beneficial effects are due to the lipid-lowering and pleiotropic properties of these compounds. Pleiotropic capacities refer to the secondary effects of medicinal treatments, such as anti-inflammatory effects and anti-oxidative stress (19, 44, 45). Various preclinical and clinical studies have reported the protective role of pleiotropic effects in lung diseases, including acute lung injury, COPD, asthma, and age-related lung diseases (27, 46–48). A recent study also provided evidence of atorvastatin and ezetimibe therapy exerting powerful cholesterol-independent effects on HCD-induced lung fibrosis in an *in vivo* model, which supports the beneficial effects of treatment with atorvastatin and ezetimibe in hypercholesterolemia-related lung diseases.

## Conclusion

Atorvastatin and ezetimibe have lipid-lowering and pleiotropic effects; therefore, atorvastatin and ezetimibe therapy can prevent hypercholesterolemia-induced pulmonary lipid oxidation, inflammation, and fibrosis in the HCD-fed rat model. The current findings appear potentially useful in the prevention of PF in a hypercholesterolemia model. The contribution of these drugs on antifibrosis is indispensable further verification in patients with dyslipidemia-related lung injury.

## Data availability statement

The original contributions presented in this study are included in the article/supplementary material, further inquiries can be directed to the corresponding author.

## Ethics statement

This study was approved by the Animal Ethics Committee of Naresuan University, Thailand (Approval number: NU-AE620718).

## Author contributions

PS, NN, and WM: conceptualization and manuscript drafting. PS, SK, TP, NN, and WM: methodology and data collection. PS, TP, and WM: data analysis. PS, SK, NN, and WM: revised the manuscript. All authors have read and approved the final manuscript.

## Abbreviations

ILD: interstitial lung disease
PF: pulmonary fibrosis
IPF: idiopathic pulmonary fibrosis
HCD: high-cholesterol diet
TC: total cholesterol
HDL-C: high-density lipoprotein cholesterol
LDL-C: low-density lipoprotein cholesterol
TG: triglyceride
TNF-α: tumor necrosis factor-alpha
IL1-β: interleukin 1-beta
IL-6: Interleukin 6
IL-10: Interleukin 10
HO-1: Heme oxygenase-1
MMP: Matrix metalloproteinase
ECM: extracellular matrix
TGF-β: transforming growth factor-beta.
